# Supplementary material for: Bile acid metabolism in multiple sclerosis is perturbed and associated with the risk of confirmed disability worsening
Source: BMC Med. 2025 Apr 9;23:212. doi: 10.1186/s12916-025-04041-x (PMC11980154; doi:10.1186/s12916-025-04041-x)
Supplement: Supplementary file 1 — Additional file 1: Tables S1-S11, Table S1. Bile acid targets and calibration, Table S2. Summary of treatments, Table S3. Description of general and clinical characteristics for the 551 PwMS included in the analysis of disability worsening, Table S4. P-values from correlation analysis for all samples (pwMS and MC), Table S5. P-values from correlation analysis for pwMS group, Table S6. P-values from correlation analysis for MC group, Table S7. Heat map data for Figure 1A, foldchanges (95% CI), and p-values, Table S8. Heat map data for Figure 1B males, foldchanges (95% CI), and p-values, Table S9. Heat map data for Figure 1B females, foldchanges (95% CI), and p-values, Table S10. EDSS 4 males and females, cox regression results. Table S11. EDSS 6 all MS, cox regression results. Figure S1-S3, Figure S1. Correlation heatmap including r-values for all samples (pwMS and MC), Figure S2. Correlation heatmap including r-values for pwMS samples, Figure S3. Correlation heatmap including r-values for MC samples. [file 12916_2025_4041_MOESM1_ESM.pdf]

Additional file 1 – Bile acid metabolism in multiple sclerosis is perturbed and associated with risk of confirmed disability worsening

Table S1 - Bile acid targets and calibration
Table S2 - Summary of treatments
Table S3 - Description of general and clinical characteristics for the 551 PwMS included in the analysis of disability worsening
Figure S1 - Correlation heatmap including r-values for all samples (pwMS and MC)
Figure S2 - Correlation heatmap including r-values for pwMS samples
Figure S3 - Correlation heatmap including r-values for MC samples
Table S4 - P-values from correlation analysis, for all samples (pwMS and MC)
Table S5 - P-values from correlation analysis, for pwMS group
Table S6 - P-values from correlation analysis, for MC group
Table S7 - Heat map data for Figure 1A, foldchanges (95% CI) and p-values.
Table S8 - Heat map data for Figure 1B males, foldchanges (95% CI) and p-values.
Table S9 - Heat map data for Figure 1B females, foldchanges (95% CI) and p-values.
Table S10 - EDSS 4 males and females, cox regression results
Table S11 - EDSS 6 all MS, cox regression results

Table S1. Summary of bile acid targets, m/z, retention time, and internal standard used for calibration.

Bile acid	***Abbreviation***	***m/z***	***RT***	***IS***
Chenodeoxycholic acid	CDCA	391.28555	8.74	TCDCA-D4
Cholic acid	CA	407.28049	8.16	TCDCA-D4
Glycochenodeoxycholic acid	GCDCA	448.30652	7.9	TCDCA-D4
Glycocholic acid	GCA	464.30148	7.37	GCA-D4
Taurochenodeoxycholic acid	TCDCA	498.28949	7.8	TCDCA-D4
Taurocholic acid	TCA	514.28412	7.25	TCA-D4
Deoxycholic acid	DCA	391.28555	8.86	TCDCA-D4
Hyocholic acid	HCA	407.28049	7.88	TCDCA-D4
Hyodeoxycholic acid	HDCA	391.28555	8.16	TCDCA-D4
Lithocholic acid	LCA	375.290525	9.33	TCDCA-D4
Muricholic acid	MCA	407.28049	7.43	GCA-D4
Murideoxycholic acid	MDCA	391.28555	7.69	TCDCA-D4
Ursodeoxycholic acid	UDCA	391.28555	7.88	TCDCA-D4
Glycodeoxycholic acid	GDCA	448.30652	8.08	TCDCA-D4
Glycohyocholic acid	GHCA	464.30148	6.91	GUDCA-D4
Glycohyodeoxycholic acid	GHCDA	448.30652	7.2	TCA-D4
Glycolithocholic acid	GLCA	432.31202	8.53	TCDCA-D4
Glycolithocholic acid 3-sulfate	GLCA-S	512.26855	7.5	GCA-D4
Glycoursodeoxycholic acid	GUDCA	448.30652	6.94	GUDCA-D4
Taurodeoxycholic acid	TDCA	498.28949	7.96	TCDCA-D4
Taurohyocholic acid	THCA	514.28412	6.8	GUDCA-D4
Taurohyodeoxycholic acid	THDCA	498.28949	7.05	GUDCA-D4
Taurolithocholic acid	TLCA	482.29453	8.4	TCDCA-D4
Tauroursodeoxycholic acid	TUDCA	498.28949	6.83	GUDCA-D4
Glycocholic acid-D4	GCA-D4	468.32687	7.37	
Glycoursodeoxycholic acid-D4	GUDCA-D4	452.33195	6.94	
Taurochenodeoxycholic acid-D4	TCDCA-D4	502.31459	7.8	
Taurocholic acid-D4	TCA-D4	518.3095	7.25	

Table S2. Treatments included in the different treatment groups

**Treatment category**	**Treatment**
First line DMT	*Teriflunomid, Interferon beta-1a, Interferon beta-1b, Glatiramer, Dimethylfumarat, Intravenous immunoglobulin (IVIG), Peginterferon beta-1a,*
Second line DMT	*Fingolimod, HSCT/BEAM, HSCT/CYK, Ofatumumab, Alemtuzumab, Kladribin, Mitoxantron, Okrelizumab, Rituximab, Natalizumab, Daklizumab beta*
Other treatments	*Erenumab, Prednison, Metylprednisolon*

Table S3. General and clinical characteristics for the 551 individuals included in the Cox proportional hazard analysis regarding confirmed disability worsening (CDW).

	RRMS	*PMS*
N	494	57
Sex (F/M)	375 / 119	39 / 18
Age		
Female (mean ± SD)	38.9±10.3	51.9±8.7
Male (mean ± SD)	37.3±10.1	48.9±8.2
BMI at sample collection		
Female (mean ± SD)	25.1±5.2	25.4±4.7
Male (mean ± SD)	25.2±4.3	25.7±2.8
Disease duration at inclusiom (months)		
Female (mean ± SD)	9.5±20.5	9.6±11.5
Male (mean ± SD)	9.3±15.1	10.5±10.3
Number of hospital visits		
Female (mean ± SD)	11.5±5.6	8.9±4.7
Male (mean ± SD)	12.3±6.7	8.9±4.6
Follow-up time (years)		
Female (mean ± SD)	10.7±3.5	9.0±4.1
Male (mean ± SD)	10.2±3.7	9.0±4.1
EDSS at the time of study inclusion		
Female (mean ± SD)	1.5±1.2	3.8±1.5
Male (mean ± SD)	2.1±1.6	3.7±1.6
EDSS milestones		
Reached EDSS 4 witin study follow-up (F/M)	64 / 32	34 / 14
Reached EDSS 6 witin study follow-up (F/M)	22 / 15	26 / 10
Treatment		
Undergoing treatment at inclusion in study (%)	8.3 %	15.8 %
First line, second line, other	25,13,3	5,3,1
Current smoking		
No. Yes/No/NA	118 / 338 / 38	9 / 44 / 4
Subjected to passive smoking		
No. Yes/No/NA	15 / 440 / 39	3 / 50 / 4
Alcohol consumption* (cl 40%)		
No. records	457	53
Female (mean ± SD)	0.9±2.9	0.6±1.6
Male (mean ± SD)	4.2±9.6	3.6±5.7
Snuff tobacco		
Current snuff users (F/M)	16 / 23	0 / 5

* *Reported intake the week leading up to blood sample collection, amount of different types of alcoholic beverages (wine, beer, liquor) recalculated to volume (cl) consumed 40% alcohol*

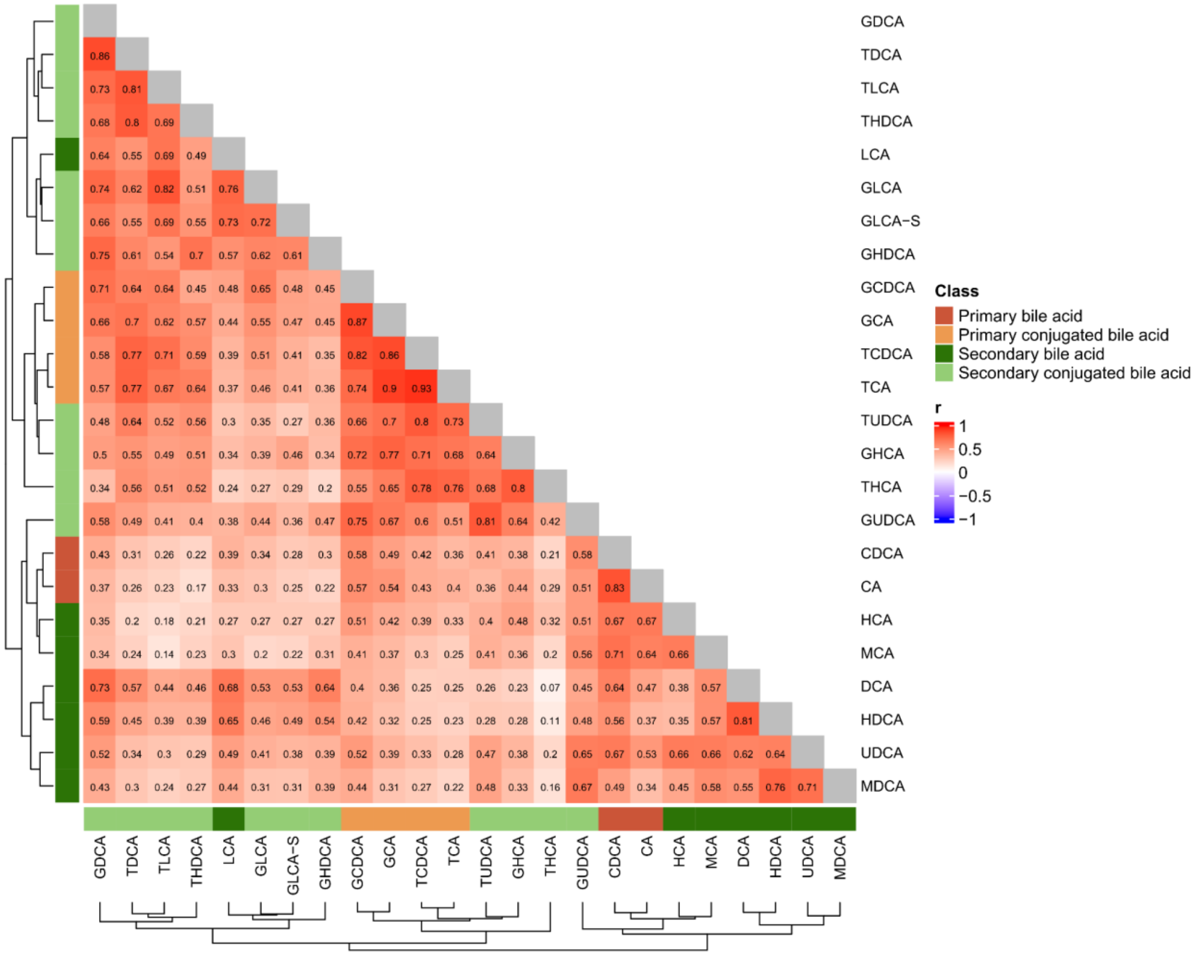

Figure S1. Correlation heat map for all BAs, including r values. The correlations are based on all samples, pwMS and MC.

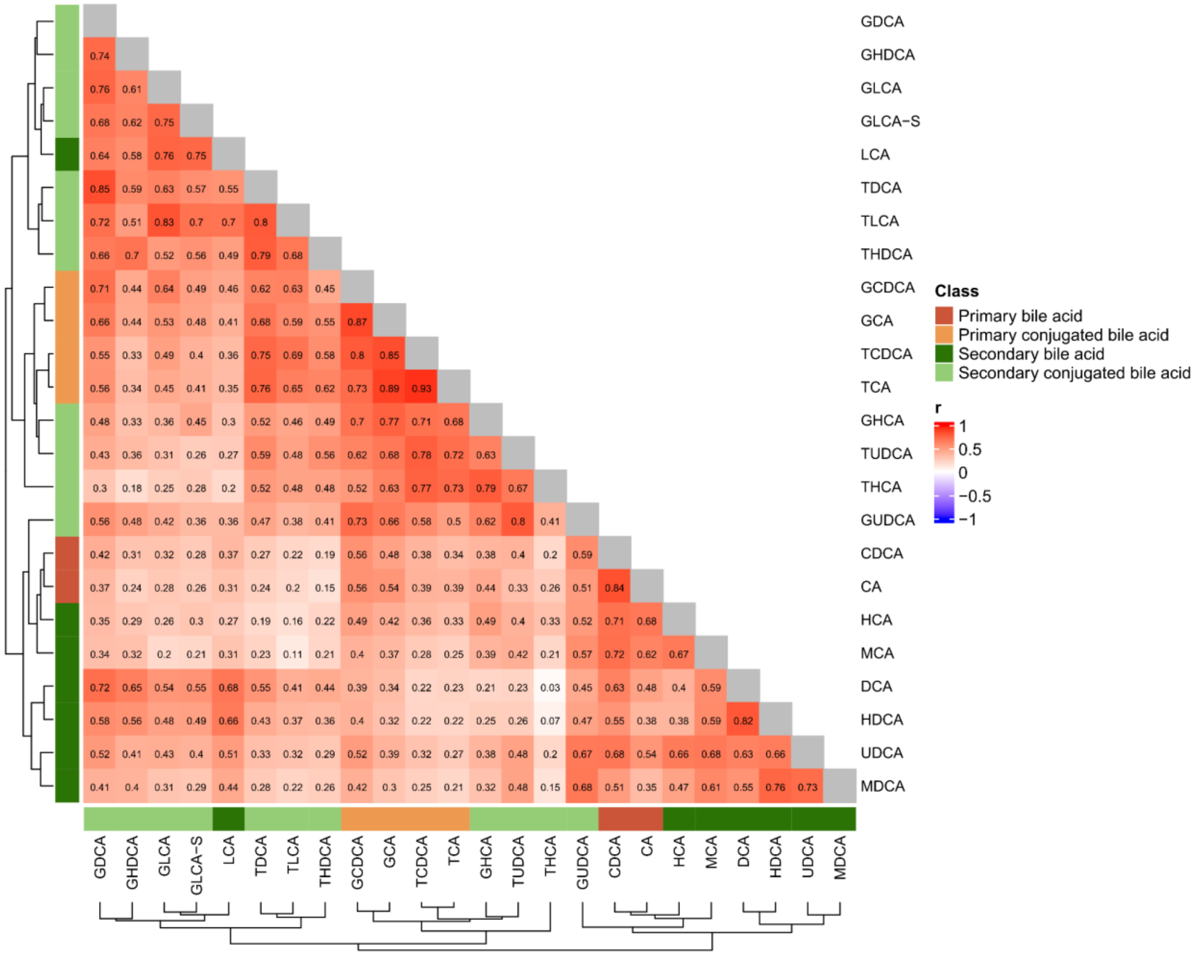

Figure S2. Correlation heat map for all BAs, including r values. The correlations are based on the pwMS group.

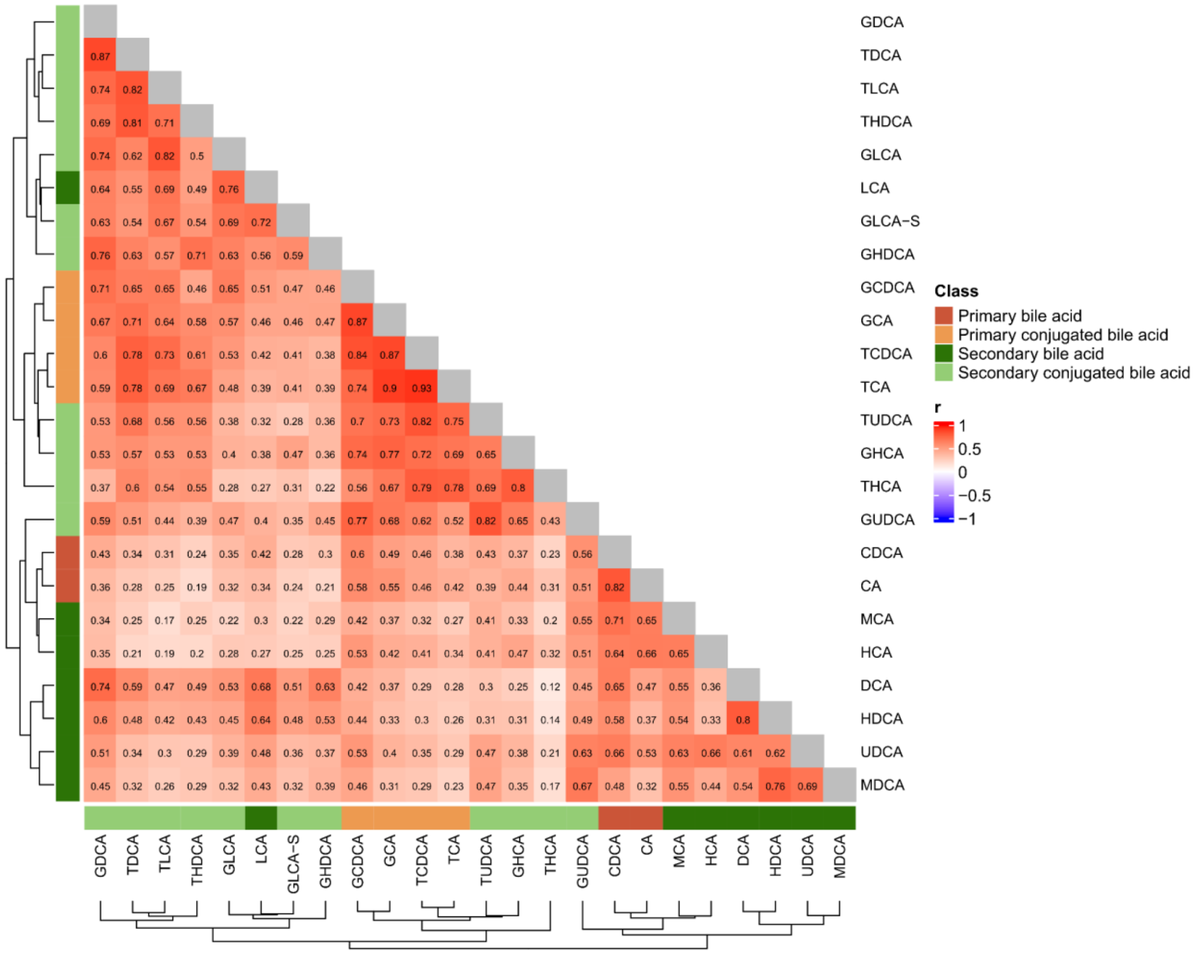

Figure S3. Correlation heat map for all BAs, including r values. The correlations are based on the MC group.

Table S4. p-values from correlation analyses (Spearman) between BAs for all samples, pwMS and MC.

	CDCA	*CA*	*GCDCA*	*GCA*	*TCDCA*	*TCA*	*DCA*	*HCA*	*HDCA*	*LCA*	*MCA*	*MDCA*	*UDCA*	*GDCA*	*GHCA*	*GHDCA*	*GLCA*	*GLCA-S*	*GUDCA*	*TDCA*	*THCA*	*THDCA*	*TLCA*	*TUDCA*
CDCA	NA	0	3.6E-162	1.2E-109	2.1E-79	1.5E-57	1.8E-206	1.3E-239	7.5E-151	3.5E-67	6.4E-243	3.8E-112	1.0E-231	1.3E-80	8.2E-63	2.9E-39	7.9E-49	9.6E-34	9.7E-160	2.0E-40	3.9E-18	1.0E-18	1.6E-29	4.9E-74
CA	0	NA	3.6E-157	5.2E-140	4.7E-81	5.0E-72	1.2E-100	2.2E-234	1.4E-60	8.7E-46	9.4E-183	5.8E-50	2.6E-132	8.2E-59	4.6E-86	1.5E-21	6.3E-39	3.8E-27	9.2E-123	9.0E-30	1.7E-32	5.2E-12	1.8E-22	7.6E-56
GCDCA	3.6E-162	3.6E-157	NA	0	0	1.2E-310	5.8E-71	4.6E-120	5.1E-77	7.1E-104	4.3E-65	7.0E-85	3.9E-127	1.1E-279	7.5E-290	5.9E-89	3.0E-214	7.5E-106	0.0	4.6E-208	8.0E-129	1.2E-83	1.6E-210	9.3E-227
GCA	1.2E-109	5.2E-140	0	NA	0	0	1.3E-55	1.4E-79	9.9E-46	2.3E-83	1.8E-51	1.1E-40	1.9E-68	1.2E-231	0	5.7E-91	1.6E-145	2.3E-100	7.0E-238	1.9E-264	1.2E-200	6.0E-141	5.0E-190	4.3E-268
TCDCA	2.1E-79	4.7E-81	0	0	NA	0	7.5E-27	6.3E-65	3.8E-28	2.2E-64	2.3E-33	2.0E-31	6.6E-48	2.2E-161	7.0E-282	3.8E-52	3.7E-121	4.8E-73	1.8E-179	0	0	1.0E-154	4.3E-280	0
TCA	1.5E-57	5.0E-72	1.2E-310	0	0	NA	5.3E-28	9.5E-48	4.2E-24	5.2E-59	9.9E-25	2.0E-20	2.3E-33	1.7E-159	1.0E-249	4.3E-57	1.2E-96	6.8E-73	5.8E-119	0	3.9E-304	8.1E-191	4.4E-237	6.1E-302
DCA	1.8E-206	1.2E-100	5.8E-71	1.3E-55	7.5E-27	5.3E-28	NA	4.9E-63	0	1.0E-240	2.4E-136	5.4E-143	3.4E-196	6.6E-299	1.1E-23	3.1E-206	9.3E-132	3.8E-131	9.7E-91	2.6E-155	3.2E-03	8.6E-86	1.5E-86	1.3E-29
HCA	1.3E-239	2.2E-234	4.6E-120	1.4E-79	6.3E-65	9.5E-48	4.9E-63	NA	1.9E-54	3.0E-31	3.4E-201	7.2E-93	5.5E-227	5.3E-54	1.8E-104	2.2E-31	7.4E-32	1.7E-32	3.7E-122	2.7E-18	3.9E-41	1.9E-17	4.2E-14	5.0E-71
HDCA	7.5E-151	1.4E-60	5.1E-77	9.9E-46	3.8E-28	4.2E-24	0	1.9E-54	NA	1.0E-216	2.7E-135	0	5.0E-208	1.1E-169	2.5E-33	1.4E-135	3.5E-96	9.0E-108	6.3E-105	5.1E-92	1.6E-05	4.7E-61	2.3E-67	1.2E-34
LCA	3.5E-67	8.7E-46	7.1E-104	2.3E-83	2.2E-64	5.2E-59	1.0E-240	3.0E-31	1.0E-216	NA	2.5E-34	2.6E-83	1.9E-110	1.5E-204	8.2E-50	6.8E-150	0	2.0E-300	3.9E-62	2.9E-141	4.9E-22	3.2E-100	4.2E-255	7.8E-38
MCA	6.4E-243	9.4E-183	4.3E-65	1.8E-51	2.3E-33	9.9E-25	2.4E-136	3.4E-201	2.7E-135	2.5E-34	NA	8.3E-143	6.2E-197	1.6E-43	5.0E-49	1.5E-35	4.4E-16	1.4E-18	2.6E-133	6.5E-22	5.8E-15	3.4E-19	3.6E-08	4.2E-65
MDCA	3.8E-112	5.8E-50	7.0E-85	1.1E-40	2.0E-31	2.0E-20	5.4E-143	7.2E-93	0	2.6E-83	8.3E-143	NA	1.0E-279	6.4E-82	9.6E-47	1.7E-66	7.5E-42	6.3E-41	1.7E-238	5.2E-38	1.3E-10	4.5E-29	3.8E-25	7.5E-102
UDCA	1.0E-231	2.6E-132	3.9E-127	1.9E-68	6.6E-48	2.3E-33	3.4E-196	5.5E-227	5.0E-208	1.9E-110	6.2E-197	1.0E-279	NA	2.5E-124	2.8E-63	9.3E-67	3.0E-73	1.0E-63	3.4E-220	1.7E-49	7.5E-17	3.4E-32	5.3E-40	2.1E-101
GDCA	1.3E-80	8.2E-59	1.1E-279	1.2E-231	2.2E-161	1.7E-159	6.6E-299	5.3E-54	1.1E-169	1.5E-204	1.6E-43	6.4E-82	2.5E-124	NA	8.4E-117	9.6E-321	3.0e-319	5.2E-225	3.8E-163	0	8.8E-45	3.0E-218	1.1E-298	8.8E-105
GHCA	8.2E-63	4.6E-86	7.5E-290	0	7.0E-282	1.0E-249	1.1E-23	1.8E-104	2.5E-33	8.2E-50	5.0E-49	9.6E-47	2.8E-63	8.4E-117	NA	5.5E-50	1.5E-65	3.6E-97	1.1E-205	1.2E-140	0	7.3E-109	2.4E-111	1.6E-207
GHDCA	2.9E-39	1.5E-21	5.9E-89	5.7E-91	3.8E-52	4.3E-57	3.1E-206	2.2E-31	1.4E-135	6.8E-150	1.5E-35	1.7E-66	9.3E-67	9.6E-321	5.5E-50	NA	6.7E-189	6.3E-181	8.4E-97	4.9E-182	6.7E-16	4.3E-239	3.6E-134	6.0E-54
GLCA	7.9E-49	6.3E-39	3.0E-214	1.6E-145	3.7E-121	1.2E-96	9.3E-132	7.4E-32	3.5E-96	0	4.4E-16	7.5E-42	3.0E-73	3.0e-319	1.5E-65	6.7E-189	NA	8.3E-283	9.4E-88	9.8E-196	7.9E-29	2.5E-107	0	2.6E-52
GLCA-S	9.6E-34	3.8E-27	7.5E-106	2.3E-100	4.8E-73	6.8E-73	3.8E-131	1.7E-32	9.0E-108	2.0E-300	1.4E-18	6.3E-41	1.0E-63	5.2E-225	3.6E-97	6.3E-181	8.3E-283	NA	1.1E-55	7.1E-147	6.4E-34	1.4E-128	6.3E-254	7.5E-32
GUDCA	9.7E-160	9.2E-123	0	7.0E-238	1.8E-179	5.8E-119	9.7E-91	3.7E-122	6.3E-105	3.9E-62	2.6E-133	1.7E-238	3.4E-220	3.8E-163	1.1E-205	8.4E-97	9.4E-88	1.1E-55	NA	7.9E-110	1.8E-71	6.6E-63	1.4E-73	0
TDCA	2.0E-40	9.0E-30	4.6E-208	1.9E-264	0	0	2.6E-155	2.7E-18	5.1E-92	2.9E-141	6.5E-22	5.2E-38	1.7E-49	0	1.2E-140	4.9E-182	9.8E-196	7.1E-147	7.9E-110	NA	6.2E-139	0	0	5.4E-205
THCA	3.9E-18	1.7E-32	8.0E-129	1.2E-200	0	3.9E-304	3.2E-03	3.9E-41	1.6E-05	4.9E-22	5.8E-15	1.3E-10	7.5E-17	8.8E-45	0	6.7E-16	7.9E-29	6.4E-34	1.8E-71	6.2E-139	NA	1.7E-103	1.8E-107	1.7E-221
THDCA	1.0E-18	5.2E-12	1.2E-83	6.0E-141	1.0E-154	8.1E-191	8.6E-86	1.9E-17	4.7E-61	3.2E-100	3.4E-19	4.5E-29	3.4E-32	3.0E-218	7.3E-109	4.3E-239	2.5E-107	1.4E-128	6.6E-63	0	1.7E-103	NA	5.2E-232	6.2E-135
TLCA	1.6E-29	1.8E-22	1.6E-210	5.0E-190	4.3E-280	4.4E-237	1.5E-86	4.2E-14	2.3E-67	4.2E-255	3.6E-08	3.8E-25	5.3E-40	1.1E-298	2.4E-111	3.6E-134	0	6.3E-254	1.4E-73	0	1.8E-107	5.2E-232	NA	9.8E-127
TUDCA	4.9E-74	7.6E-56	9.3E-227	4.3E-268	0	6.1E-302	1.3E-29	5.0E-71	1.2E-34	7.8E-38	4.2E-65	7.5E-102	2.1E-101	8.8E-105	1.6E-207	6.0E-54	2.6E-52	7.5E-32	0	5.4E-205	1.7E-221	6.2E-135	9.8E-127	NA

Table S5. p-values from correlation analyses (Spearman) between BAs for pwMS group.

	CDCA	*CA*	*GCDCA*	*GCA*	*TCDCA*	*TCA*	*DCA*	*HCA*	*HDCA*	*LCA*	*MCA*	*MDCA*	*UDCA*	*GDCA*	*GHCA*	*GHDCA*	*GLCA*	*GLCA-S*	*GUDCA*	*TDCA*	*THCA*	*THDCA*	*TLCA*	*TUDCA*
CDCA	NA	0	3.6E-162	1.2E-109	2.1E-79	1.5E-57	1.8E-206	1.3E-239	7.5E-151	3.5E-67	6.4E-243	3.8E-112	1.0E-231	1.3E-80	8.2E-63	2.9E-39	7.9E-49	9.6E-34	9.7E-160	2.0E-40	3.9E-18	1.0E-18	1.6E-29	4.9E-74
CA	0	NA	3.6E-157	5.2E-140	4.7E-81	5.0E-72	1.2E-100	2.2E-234	1.4E-60	8.7E-46	9.4E-183	5.8E-50	2.6E-132	8.2E-59	4.6E-86	1.5E-21	6.3E-39	3.8E-27	9.2E-123	9.0E-30	1.7E-32	5.2E-12	1.8E-22	7.6E-56
GCDCA	3.6E-162	3.6E-157	NA	0	0	1.23E-310	5.8E-71	4.6E-120	5.1E-77	7.1E-104	4.3E-65	7.0E-85	3.9E-127	1.1E-279	7.5E-290	5.9E-89	3.0E-214	7.5E-106	0	4.6E-208	8.0E-129	1.2E-83	1.6E-210	9.3E-227
GCA	1.2E-109	5.2E-140	0	NA	0	0	1.3E-55	1.4E-79	9.9E-46	2.3E-83	1.8E-51	1.1E-40	1.9E-68	1.2E-231	0	5.7E-91	1.6E-145	2.3E-100	7.0E-238	1.9E-264	1.2E-200	6.0E-141	5.0E-190	4.3E-268
TCDCA	2.1E-79	4.7E-81	0	0	NA	0	7.5E-27	6.3E-65	3.8E-28	2.2E-64	2.3E-33	2.0E-31	6.6E-48	2.2E-161	7.0E-282	3.8E-52	3.7E-121	4.8E-73	1.8E-179	0	0	1.0E-154	4.3E-280	0
TCA	1.5E-57	5.0E-72	1.2E-310	0	0	NA	5.3E-28	9.5E-48	4.2E-24	5.2E-59	9.9E-25	2.0E-20	2.3E-33	1.7E-159	1.0E-249	4.3E-57	1.2E-96	6.8E-73	5.8E-119	0	3.9E-304	8.1E-191	4.4E-237	6.1E-302
DCA	1.8E-206	1.2E-100	5.8E-71	1.3E-55	7.5E-27	5.3E-28	NA	4.9E-63	0	1.0E-240	2.4E-136	5.4E-143	3.4E-196	6.6E-299	1.1E-23	3.1E-206	9.3E-132	3.8E-131	9.7E-91	2.6E-155	3.2E-03	8.6E-86	1.5E-86	1.3E-29
HCA	1.3E-239	2.2E-234	4.6E-120	1.4E-79	6.3E-65	9.5E-48	4.9E-63	NA	1.9E-54	3.0E-31	3.4E-201	7.2E-93	5.5E-227	5.3E-54	1.8E-104	2.2E-31	7.4E-32	1.7E-32	3.7E-122	2.7E-18	3.9E-41	1.9E-17	4.2E-14	5.0E-71
HDCA	7.5E-151	1.4E-60	5.1E-77	9.9E-46	3.8E-28	4.2E-24	0	1.9E-54	NA	1.0E-216	2.7E-135	0	5.0E-208	1.1E-169	2.5E-33	1.4E-135	3.5E-96	9.0E-108	6.3E-105	5.1E-92	1.6E-05	4.7E-61	2.3E-67	1.2E-34
LCA	3.5E-67	8.7E-46	7.1E-104	2.3E-83	2.2E-64	5.2E-59	1.0E-240	3.0E-31	1.0E-216	NA	2.5E-34	2.6E-83	1.9E-110	1.5E-204	8.2E-50	6.8E-150	0	2.0E-300	3.9E-62	2.9E-141	4.9E-22	3.2E-100	4.2E-255	7.8E-38
MCA	6.4E-243	9.4E-183	4.3E-65	1.8E-51	2.3E-33	9.9E-25	2.4E-136	3.4E-201	2.7E-135	2.5E-34	NA	8.3E-143	6.2E-197	1.6E-43	5.0E-49	1.5E-35	4.4E-16	1.4E-18	2.6E-133	6.5E-22	5.8E-15	3.4E-19	3.6E-08	4.2E-65
MDCA	3.8E-112	5.8E-50	7.0E-85	1.1E-40	2.0E-31	2.0E-20	5.4E-143	7.2E-93	0	2.6E-83	8.3E-143	NA	1.0E-279	6.4E-82	9.6E-47	1.7E-66	7.5E-42	6.3E-41	1.7E-238	5.2E-38	1.3E-10	4.5E-29	3.8E-25	7.5E-102
UDCA	1.0E-231	2.6E-132	3.9E-127	1.9E-68	6.6E-48	2.3E-33	3.4E-196	5.5E-227	5.0E-208	1.9E-110	6.2E-197	1.0E-279	NA	2.5E-124	2.8E-63	9.3E-67	3.0E-73	1.0E-63	3.4E-220	1.7E-49	7.5E-17	3.4E-32	5.3E-40	2.1E-101
GDCA	1.3E-80	8.2E-59	1.1E-279	1.2E-231	2.2E-161	1.7E-159	6.6E-299	5.3E-54	1.1E-169	1.5E-204	1.6E-43	6.4E-82	2.5E-124	NA	8.4E-117	9.6E-321	3.0e-319	5.2E-225	3.8E-163	0	8.8E-45	3.0E-218	1.1E-298	8.8E-105
GHCA	8.2E-63	4.6E-86	7.5E-290	0	7.0E-282	1.0E-249	1.1E-23	1.8E-104	2.5E-33	8.2E-50	5.0E-49	9.6E-47	2.8E-63	8.4E-117	NA	5.5E-50	1.5E-65	3.6E-97	1.1E-205	1.2E-140	0	7.3E-109	2.4E-111	1.6E-207
GHDCA	2.9E-39	1.5E-21	5.9E-89	5.7E-91	3.8E-52	4.3E-57	3.1E-206	2.2E-31	1.4E-135	6.8E-150	1.5E-35	1.7E-66	9.3E-67	9.6E-321	5.5E-50	NA	6.7E-189	6.3E-181	8.4E-97	4.9E-182	6.7E-16	4.3E-239	3.6E-134	6.0E-54
GLCA	7.9E-49	6.3E-39	3.0E-214	1.6E-145	3.7E-121	1.2E-96	9.3E-132	7.4E-32	3.5E-96	0	4.4E-16	7.5E-42	3.0E-73	3.0e-319	1.5E-65	6.7E-189	NA	8.3E-283	9.4E-88	9.8E-196	7.9E-29	2.5E-107	0	2.6E-52
GLCA-S	9.6E-34	3.8E-27	7.5E-106	2.3E-100	4.8E-73	6.8E-73	3.8E-131	1.7E-32	9.0E-108	2.0E-300	1.4E-18	6.3E-41	1.0E-63	5.2E-225	3.6E-97	6.3E-181	8.3E-283	NA	1.1E-55	7.1E-147	6.4E-34	1.4E-128	6.3E-254	7.5E-32
GUDCA	9.7E-160	9.2E-123	0	7.0E-238	1.8E-179	5.8E-119	9.7E-91	3.7E-122	6.3E-105	3.9E-62	2.6E-133	1.7E-238	3.4E-220	3.8E-163	1.1E-205	8.4E-97	9.4E-88	1.1E-55	NA	7.9E-110	1.8E-71	6.6E-63	1.4E-73	0
TDCA	2.0E-40	9.0E-30	4.6E-208	1.9E-264	0	0	2.6E-155	2.7E-18	5.1E-92	2.9E-141	6.5E-22	5.2E-38	1.7E-49	0	1.2E-140	4.9E-182	9.8E-196	7.1E-147	7.9E-110	NA	6.2E-139	0	0	5.4E-205
THCA	3.9E-18	1.7E-32	8.0E-129	1.2E-200	0	3.9E-304	3.2E-03	3.9E-41	1.6E-05	4.9E-22	5.8E-15	1.3E-10	7.5E-17	8.8E-45	0	6.7E-16	7.9E-29	6.4E-34	1.8E-71	6.2E-139	NA	1.7E-103	1.8E-107	1.7E-221
THDCA	1.0E-18	5.2E-12	1.2E-83	6.0E-141	1.0E-154	8.1E-191	8.6E-86	1.9E-17	4.7E-61	3.2E-100	3.4E-19	4.5E-29	3.4E-32	3.0E-218	7.3E-109	4.3E-239	2.5E-107	1.4E-128	6.6E-63	0	1.7E-103	NA	5.2E-232	6.2E-135
TLCA	1.6E-29	1.8E-22	1.6E-210	5.0E-190	4.3E-280	4.4E-237	1.5E-86	4.2E-14	2.3E-67	4.2E-255	3.6E-08	3.8E-25	5.3E-40	1.1E-298	2.4E-111	3.6E-134	0	6.3E-254	1.4E-73	0	1.8E-107	5.2E-232	NA	9.8E-127
TUDCA	4.9E-74	7.6E-56	9.3E-227	4.3E-268	0	6.1E-302	1.3E-29	5.0E-71	1.2E-34	7.8E-38	4.2E-65	7.5E-102	2.1E-101	8.8E-105	1.6E-207	6.0E-54	2.6E-52	7.5E-32	0	5.4E-205	1.7E-221	6.2E-135	9.8E-127	NA

Table S6. p-values from correlation analyses (Spearman) between BAs for MC group.

	CDCA	*CA*	*GCDCA*	*GCA*	*TCDCA*	*TCA*	*DCA*	*HCA*	*HDCA*	*LCA*	*MCA*	*MDCA*	*UDCA*	*GDCA*	*GHCA*	*GHDCA*	*GLCA*	*GLCA-S*	*GUDCA*	*TDCA*	*THCA*	*THDCA*	*TLCA*	*TUDCA*
CDCA	NA	1.5E-216	2.46E-88	5.77E-57	3.21E-48	2.41E-32	2.1E-109	1.6E-106	2.97E-82	1.39E-39	3.7E-122	5.06E-53	1.8E-112	1.83E-42	3.89E-31	6.15E-20	8.24E-28	5.39E-18	2.57E-77	1.49E-25	5.6E-11	5.21E-12	7.83E-21	6.65E-41
CA	1.5E-216	NA	2.05E-83	8.41E-73	3.72E-48	8.45E-40	1.98E-50	5E-113	2.48E-30	6.54E-26	6.09E-98	1.06E-23	1.52E-65	2.2E-29	1.83E-43	3.92E-10	1.14E-22	2.01E-13	3.07E-62	7.39E-18	1.49E-19	8.92E-08	1.69E-14	2.0E-33
GCDCA	2.46E-88	2.05E-83	NA	2.1E-284	2.7E-241	3E-159	7.77E-40	3.79E-66	1.54E-44	9.96E-60	3.1E-36	3.43E-49	7.07E-68	4.4E-141	8.8E-156	1.1E-47	8E-110	2.94E-50	5.7E-176	5.9E-110	4.11E-71	3.83E-43	9.3E-110	2.1E-131
GCA	5.77E-57	8.41E-73	2.1E-284	NA	1.5E-277	0	3.52E-31	7.46E-41	3.56E-25	6.26E-49	8.86E-28	4.96E-22	1.73E-35	1.3E-118	2.1E-176	2.16E-50	8.11E-78	1.93E-49	5.6E-122	1.8E-139	1.2E-109	2.95E-75	1E-104	4.6E-149
TCDCA	3.21E-48	3.72E-48	2.7E-241	1.5E-277	NA	0	1.97E-18	2.02E-37	5.79E-20	5.31E-39	4.89E-21	1.4E-19	3.16E-28	3.72E-91	5.6E-146	2.32E-31	6.12E-66	2.92E-38	2.51E-98	5.5E-187	5.1E-179	3.26E-83	4.4E-151	1.4E-223
TCA	2.41E-32	8.45E-40	3E-159	0	0	NA	1.48E-17	2.29E-25	2.66E-15	2.21E-33	1.11E-14	6.17E-12	8.86E-19	3.68E-86	7.2E-127	1.2E-33	2.54E-52	1.45E-37	6.21E-63	3E-185	3.1E-168	2.7E-104	3.5E-129	7E-163
DCA	2.1E-109	1.98E-50	7.77E-40	3.52E-31	1.97E-18	1.48E-17	NA	2.55E-28	1.8E-205	1.7E-121	1.68E-63	2.64E-70	5.88E-95	1.6E-155	8.66E-15	1.1E-100	4.82E-67	3.32E-60	1.72E-47	1.89E-85	0.00082	4.15E-49	2.01E-51	9.64E-20
HCA	1.6E-106	5E-113	3.79E-66	7.46E-41	2.02E-37	2.29E-25	2.55E-28	NA	4.91E-24	7.3E-17	2.15E-98	5.07E-43	1.8E-113	2.01E-27	1.02E-49	3.46E-14	1.08E-17	5.77E-14	1.48E-60	1.62E-10	5.05E-21	1.21E-08	6.06E-09	3.87E-37
HDCA	2.97E-82	2.48E-30	1.54E-44	3.56E-25	5.79E-20	2.66E-15	1.8E-205	4.91E-24	NA	2.1E-105	1.35E-60	3.4E-172	6.87E-96	1.63E-88	1.54E-21	1.5E-64	5.31E-47	2.99E-54	3.03E-55	1.19E-52	5.17E-05	4.06E-38	3.38E-39	5.96E-22
LCA	1.39E-39	6.54E-26	9.96E-60	6.26E-49	5.31E-39	2.21E-33	1.7E-121	7.3E-17	2.1E-105	NA	1.21E-17	2.86E-41	1.8E-53	7E-103	6.8E-33	5.31E-73	1.8E-166	1E-143	2.27E-35	3.39E-72	1.33E-15	1.33E-50	7.5E-128	6.23E-23
MCA	3.7E-122	6.09E-98	3.1E-36	8.86E-28	4.89E-21	1.11E-14	1.68E-63	2.15E-98	1.35E-60	1.21E-17	NA	8.6E-65	1.89E-90	4.17E-23	2.47E-22	1.29E-16	5.05E-10	1.25E-10	4.42E-65	6.08E-13	4.04E-08	3.29E-12	1.02E-06	5.12E-33
MDCA	5.06E-53	1.06E-23	3.43E-49	4.96E-22	1.4E-19	6.17E-12	2.64E-70	5.07E-43	3.4E-172	2.86E-41	8.6E-65	NA	4.5E-130	6.23E-47	6.78E-27	2.14E-33	5.52E-23	2.13E-23	2.8E-119	1.11E-22	9.19E-07	2.51E-17	1.33E-15	7.67E-52
UDCA	1.8E-112	1.52E-65	7.07E-68	1.73E-35	3.16E-28	8.86E-19	5.88E-95	1.8E-113	6.87E-96	1.8E-53	1.89E-90	4.5E-130	NA	8.38E-62	1.91E-32	5.14E-31	6.06E-35	6.29E-29	5.4E-103	4.77E-26	1.96E-09	4.3E-17	1.09E-19	3.94E-50
GDCA	1.83E-42	2.2E-29	4.4E-141	1.3E-118	3.72E-91	3.68E-86	1.6E-155	2.01E-27	1.63E-88	7E-103	4.17E-23	6.23E-47	8.38E-62	NA	1.33E-66	2.9E-168	3.3E-156	2.8E-102	2.47E-87	7E-276	5.86E-29	2E-114	2.1E-158	1.27E-65
GHCA	3.89E-31	1.83E-43	8.8E-156	2.1E-176	5.6E-146	7.2E-127	8.66E-15	1.02E-49	1.54E-21	6.8E-33	2.47E-22	6.78E-27	1.91E-32	1.33E-66	NA	8.43E-29	5.61E-37	4.67E-52	7.7E-109	4.94E-80	1.3E-184	7.47E-59	3.89E-65	1.3E-110
GHDCA	6.15E-20	3.92E-10	1.1E-47	2.16E-50	2.32E-31	1.2E-33	1.1E-100	3.46E-14	1.5E-64	5.31E-73	1.29E-16	2.14E-33	5.14E-31	2.9E-168	8.43E-29	NA	3.9E-101	3.84E-85	6.06E-46	3E-99	1.14E-10	8.5E-122	1.78E-77	3.66E-28
GLCA	8.24E-28	1.14E-22	8E-110	8.11E-78	6.12E-66	2.54E-52	4.82E-67	1.08E-17	5.31E-47	1.8E-166	5.05E-10	5.52E-23	6.06E-35	3.3E-156	5.61E-37	3.9E-101	NA	1.2E-127	9.57E-50	3.95E-96	9.2E-17	4.33E-52	2.1E-218	1.45E-32
GLCA-S	5.39E-18	2.01E-13	2.94E-50	1.93E-49	2.92E-38	1.45E-37	3.32E-60	5.77E-14	2.99E-54	1E-143	1.25E-10	2.13E-23	6.29E-29	2.8E-102	4.67E-52	3.84E-85	1.2E-127	NA	7.51E-28	2.95E-69	9.44E-20	1.85E-62	1.5E-120	8.13E-18
GUDCA	2.57E-77	3.07E-62	5.7E-176	5.6E-122	2.51E-98	6.21E-63	1.72E-47	1.48E-60	3.03E-55	2.27E-35	4.42E-65	2.8E-119	5.4E-103	2.47E-87	7.7E-109	6.06E-46	9.57E-50	7.51E-28	NA	9.56E-61	1.35E-39	1.15E-30	4.74E-43	7.4E-219
TDCA	1.49E-25	7.39E-18	5.9E-110	1.8E-139	5.5E-187	3E-185	1.89E-85	1.62E-10	1.19E-52	3.39E-72	6.08E-13	1.11E-22	4.77E-26	7E-276	4.94E-80	3E-99	3.95E-96	2.95E-69	9.56E-61	NA	4.27E-84	6.8E-190	1.4E-223	2.3E-121
THCA	5.6E-11	1.49E-19	4.11E-71	1.2E-109	5.1E-179	3.1E-168	0.00082	5.05E-21	5.17E-05	1.33E-15	4.04E-08	9.19E-07	1.96E-09	5.86E-29	1.3E-184	1.14E-10	9.2E-17	9.44E-20	1.35E-39	4.27E-84	NA	4.19E-62	9.98E-63	1.8E-117
THDCA	5.21E-12	8.92E-08	3.83E-43	2.95E-75	3.26E-83	2.7E-104	4.15E-49	1.21E-08	4.06E-38	1.33E-50	3.29E-12	2.51E-17	4.3E-17	2E-114	7.47E-59	8.5E-122	4.33E-52	1.85E-62	1.15E-30	6.8E-190	4.19E-62	NA	1E-123	3.27E-67
TLCA	7.83E-21	1.69E-14	9.3E-110	1E-104	4.4E-151	3.5E-129	2.01E-51	6.06E-09	3.38E-39	7.5E-128	1.02E-06	1.33E-15	1.09E-19	2.1E-158	3.89E-65	1.78E-77	2.1E-218	1.5E-120	4.74E-43	1.4E-223	9.98E-63	1E-123	NA	2.86E-76
TUDCA	6.65E-41	2.0E-33	2.1E-131	4.6E-149	1.4E-223	7E-163	9.64E-20	3.87E-37	5.96E-22	6.23E-23	5.12E-33	7.67E-52	3.94E-50	1.27E-65	1.3E-110	3.66E-28	1.45E-32	8.13E-18	7.4E-219	2.3E-121	1.8E-117	3.27E-67	2.86E-76	NA

Table S7. $\log_2$ fold changes, including 95% confidence intervals and p-values for comparisons of all PwMS and matched controls shown in Figure 1A. A p-value ≤0.0125 was considered significant, significant p-values are highlighted with bold text.

	MS vs MC		***RRMS vs MC***		***PMS vs MC***	
	log₂ FC (95% CI)	***p-value***	***log₂ FC (95% CI)***	***p-value***	***log₂ FC (95% CI)***	***p-value***
CDCA	-0.332 (-0.662 - -0.003)	0.048	-0.205 (-0.435 - 0.025)	0.081	-0.396 (-0.857 - 0.064)	0.092
CA	-0.153 (-0.496 - 0.19)	0.382	-0.085 (-0.326 - 0.156)	0.489	-0.187 (-0.665 - 0.291)	0.443
Primary non-conjugated	-0.322 (-0.638 - -0.006)	0.046	-0.219 (-0.440 - 0.001)	0.051	-0.373 (-0.814 - 0.069)	0.098
GCDCA	**-0.291 (-0.510 - -0.073)**	**9.06E-03**	**-0.302 (-0.451 - -0.153)**	**7.88E-05**	-0.286 (-0.594 - 0.022)	0.069
GCA	-0.261 (-0.499 - -0.023)	0.032	**-0.297 (-0.460 - -0.134)**	**3.76E-04**	-0.243 (-0.578 - 0.093)	0.156
TCDCA	-0.295 (-0.560 - -0.030)	0.029	**-0.351 (-0.534 - -0.168)**	**1.83E-04**	-0.267 (-0.639 - 0.105)	0.159
TCA	-0.242 (-0.555 - 0.070)	0.128	**-0.332 (-0.549 - -0.114)**	**2.87E-03**	-0.198 (-0.634 - 0.239)	0.374
Primary conjugated	-0.265 (-0.483 - -0.046)	0.018	**-0.301 (-0.451 - -0.152)**	**8.40E-05**	-0.246 (-0.555 - 0.062)	0.118
DCA	-0.058 (-0.374 - 0.258)	0.719	0.030 (-0.189 - 0.249)	0.788	-0.102 (-0.544 - 0.340)	0.651
HCA	-0.126 (-0.468 - 0.216)	0.470	-0.213 (-0.451 - 0.024)	0.079	-0.082 (-0.562 - 0.397)	0.736
HDCA	-0.089 (-0.339 - 0.161)	0.485	0.140 (-0.032 - 0.312)	0.111	-0.204 (-0.556 - 0.149)	0.257
LCA	-0.165 (-0.410 - 0.081)	0.188	-0.003 (-0.172 - 0.165)	0.968	-0.245 (-0.590 - 0.099)	0.163
MCA	0.046 (-0.260 - 0.351)	0.769	0.175 (-0.039 - 0.388)	0.109	-0.019 (-0.447 - 0.409)	0.931
MDCA	-0.149 (-0.444 - 0.147)	0.324	0.115 (-0.093 - 0.323)	0.278	-0.281 (-0.692 - 0.131)	0.181
UDCA	-0.086 (-0.377 - 0.206)	0.564	0.030 (-0.173 - 0.233)	0.774	-0.143 (-0.550 - 0.264)	0.490
Secondary non-conjugated	-0.101 (-0.336 - 0.133)	0.397	0.048 (-0.113 - 0.208)	0.562	-0.176 (-0.507 - 0.155)	0.297
GDCA	-0.041 (-0.379 - 0.297)	0.813	-0.129 (-0.367 - 0.108)	0.286	0.003 (-0.467 - 0.473)	0.989
GHCA	-0.246 (-0.459 - -0.034)	0.023	**-0.259 (-0.403 - -0.114)**	**4.75E-04**	-0.240 (-0.540 - 0.060)	0.116
GHDCA	-0.103 (-0.469 - 0.262)	0.579	-0.050 (-0.305 - 0.206)	0.704	-0.130 (-0.641 - 0.380)	0.617
GLCA	-0.433 (-0.784 - -0.081)	0.016	**-0.359 (-0.606 - -0.113)**	**4.35E-03**	-0.470 (-0.960 - 0.020)	0.060
GLCA-S	0.030 (-0.208 - 0.268)	0.803	0.030 (-0.133 - 0.193)	0.717	0.030 (-0.305 - 0.366)	0.859
GUDCA	-0.221 (-0.473 - 0.031)	0.086	-0.206 (-0.38 - -0.033)	0.020	-0.228 (-0.583 - 0.126)	0.206
TDCA	-0.122 (-0.468 - 0.224)	0.489	-0.226 (-0.47 - 0.017)	0.069	-0.070 (-0.550 - 0.411)	0.776
THCA	-0.161 (-0.526 - 0.204)	0.386	-0.252 (-0.508 - 0.003)	0.053	-0.116 (-0.626 - 0.395)	0.657
THDCA	-0.148 (-0.522 - 0.227)	0.439	-0.305 (-0.571 - -0.039)	0.025	-0.069 (-0.59 - 0.452)	0.795
TLCA	-0.193 (-0.501 - 0.116)	0.221	**-0.282 (-0.497 - -0.067)**	**0.010**	-0.148 (-0.579 - 0.283)	0.501
TUDCA	-0.192 (-0.505 - 0.122)	0.230	-0.265 (-0.483 - -0.047)	0.017	-0.155 (-0.594 - 0.284)	0.489
Secondary conjugated	-0.071 (-0.267 - 0.126)	0.480	**-0.184 (-0.317 - -0.052)**	**6.59E-03**	-0.014 (-0.291 - 0.264)	0.923
Total bile acids	-0.148 (-0.325 - 0.028)	0.099	**-0.220 (-0.339 - -0.101)**	**2.99E-04**	-0.113 (-0.363 - 0.138)	0.378

Table S8. $\log_2$ fold changes, including 95% confidence intervals and p-values for comparisons of **male** PwMS and matched controls shown in Figure 1B. A p-value ≤0.0125 was considered significant, significant p-values are highlighted with bold text.

	MS vs MC		***RRMS vs MC***		***PMS vs MC***	
	$\log_2$ FC (95% CI)	***p-value***	***$\log_2$ FC (95% CI)***	***p-value***	***$\log_2$ FC (95% CI)***	***p-value***
CDCA	-0.469 (-0.989 - 0.051)	0.077	-0.316 (-0.712 - 0.08)	0.118	-0.546 (-1.26 - 0.171)	0.136
CA	-0.219 (-0.761 - 0.324)	0.429	-0.264 (-0.679 - 0.151)	0.212	-0.196 (-0.942 - 0.55)	0.607
Primary non-conjugated	-0.470 (-0.814 - -0.125)	0.065	-0.432 (-0.689 - -0.175)	0.040	-0.488 (-0.97 - -0.007)	0.150
GCDCA	**-0.481 (-0.857 - -0.106)**	**7.66E-03**	**-0.486 (-0.767 - -0.206)**	**1.02E-03**	-0.479 (-1.00 - 0.045)	0.047
GCA	**-0.486 (-0.905 - -0.068)**	**0.012**	**-0.529 (-0.845 - -0.214)**	**6.97E-04**	-0.464 (-1.05 - 0.117)	0.073
TCDCA	-0.435 (-0.93 - 0.061)	0.023	**-0.515 (-0.890 - -0.141)**	**1.02E-03**	-0.394 (-1.08 - 0.289)	0.117
TCA	-0.351 (-0.850 - 0.147)	0.085	**0.025 (-0.352 - 0.402)**	**7.05E-03**	-0.539 (-1.23 - 0.151)	0.258
Primary conjugated	**-0.424 (-0.964 - 0.116)**	**9.08E-03**	**-0.474 (-0.883 - -0.065)**	**4.25E-04**	-0.399 (-1.15 - 0.35)	0.063
DCA	-0.215 (-0.610 - 0.180)	0.167	0.263 (-0.033 - 0.559)	0.896	-0.454 (-1.004 - 0.096)	0.126
HCA	-0.357 (-0.742 - 0.028)	0.124	0.097 (-0.193 - 0.386)	0.023	-0.584 (-1.119 - -0.048)	0.296
HDCA	-0.295 (-0.762 - 0.173)	0.286	0.218 (-0.148 - 0.583)	0.081	-0.551 (-1.197 - 0.095)	0.106
LCA	-0.364 (-0.831 - 0.104)	0.069	0.205 (-0.153 - 0.564)	0.512	-0.649 (-1.29 - -0.007)	0.033
MCA	-0.402 (-0.862 - 0.058)	0.216	0.021 (-0.328 - 0.371)	0.243	-0.613 (-1.249 - 0.022)	0.095
MDCA	-0.310 (-0.844 - 0.225)	0.127	-0.172 (-0.581 - 0.237)	0.261	-0.378 (-1.11 - 0.356)	0.048
UDCA	-0.460 (-0.795 - -0.125)	0.087	-0.432 (-0.681 - -0.184)	0.905	-0.474 (-0.944 - -0.005)	0.059
Secondary non-conjugated	-0.383 (-0.961 - 0.196)	0.075	-0.162 (-0.602 - 0.278)	0.562	-0.493 (-1.29 - 0.304)	0.039
GDCA	-0.728 (-1.28 - -0.172)	0.256	-0.344 (-0.768 - 0.080)	0.408	-0.92 (-1.69 - -0.154)	0.312
GHCA	**-0.028 (-0.404 - 0.347)**	**7.10E-03**	**0.134 (-0.146 - 0.414)**	**6.79E-04**	-0.109 (-0.633 - 0.415)	0.048
GHDCA	-0.447 (-0.845 - -0.049)	0.194	-0.394 (-0.693 - -0.095)	0.471	-0.474 (-1.03 - 0.079)	0.225
GLCA	**-0.369 (-0.916 - 0.177)**	**0.010**	-0.306 (-0.725 - 0.113)	0.112	-0.401 (-1.15 - 0.349)	0.019
GLCA-S	-0.394 (-0.967 - 0.179)	0.883	-0.465 (-0.906 - -0.024)	0.348	-0.358 (-1.15 - 0.433)	0.682
GUDCA	-0.474 (-1.07 - 0.120)	0.028	**-0.479 (-0.936 - -0.022)**	**0.010**	-0.471 (-1.29 - 0.346)	0.093
TDCA	-0.456 (-0.944 - 0.033)	0.185	-0.403 (-0.773 - -0.033)	0.152	-0.482 (-1.16 - 0.192)	0.295
THCA	-0.439 (-0.935 - 0.057)	0.178	-0.579 (-0.954 - -0.203)	0.039	-0.369 (-1.06 - 0.319)	0.375
THDCA	-0.342 (-0.62 - -0.064)	0.118	-0.334 (-0.539 - -0.129)	0.040	-0.346 (-0.738 - 0.046)	0.258
TLCA	-0.470 (-0.969 - 0.030)	0.068	-0.398 (-0.777 - -0.018)	0.033	-0.506 (-1.20 - 0.184)	0.161
TUDCA	-0.336 (-0.707 - 0.034)	0.083	**0.082 (-0.195 - 0.359)**	**2.55E-03**	-0.546 (-1.06 - -0.029)	0.293
Secondary conjugated	-0.460 (-0.805 - -0.115)	0.057	-0.464 (-0.721 - -0.207)	0.021	-0.458 (-0.941 - 0.025)	0.154
Total bile acids	-0.300 (-0.609 - 0.009)	0.016	**-0.269 (-0.497 - -0.040)**	**1.41E-03**	-0.316 (-0.750 - 0.119)	0.084

Table S9. $\log_2$ fold changes, including 95% confidence intervals and p-values for comparisons of **female** PwMS and matched controls shown in Figure 1B. A p-value ≤0.0125 was considered significant, significant p-values are highlighted with bold text.

	MS vs MC		***RRMS vs MC***		***PMS vs MC***	
	FC (95% CI)	***p-value***	***FC (95% CI)***	***p-value***	***FC (95% CI)***	***p-value***
CDCA	-0.195 (-0.582 - 0.191)	0.321	-0.094 (-0.316 - 0.129)	0.410	-0.246 (-0.798 - 0.305)	0.381
CA	-0.087 (-0.487 - 0.313)	0.669	0.094 (-0.140 - 0.328)	0.431	-0.178 (-0.747 - 0.392)	0.540
Primary non-conjugated	-0.174 (-0.542 - 0.195)	0.356	-0.041 (-0.255 - 0.172)	0.704	-0.240 (-0.766 - 0.287)	0.372
GCDCA	-0.113 (-0.369 - 0.143)	0.387	-0.172 (-0.317 - -0.028)	0.019	-0.083 (-0.452 - 0.285)	0.658
GCA	-0.040 (-0.319 - 0.239)	0.779	-0.107 (-0.264 - 0.051)	0.183	-0.006 (-0.408 - 0.395)	0.975
TCDCA	-0.104 (-0.414 - 0.206)	0.510	-0.172 (-0.349 - 0.005)	0.057	-0.070 (-0.515 - 0.374)	0.756
TCA	-0.050 (-0.413 - 0.313)	0.786	-0.148 (-0.359 - 0.063)	0.170	-0.002 (-0.520 - 0.516)	0.995
Primary conjugated	-0.069 (-0.326 - 0.188)	0.598	-0.138 (-0.283 - 0.006)	0.060	-0.034 (-0.404 - 0.335)	0.855
DCA	0.235 (-0.134 - 0.605)	0.212	0.035 (-0.177 - 0.247)	0.747	0.335 (-0.193 - 0.864)	0.213
HCA	0.172 (-0.228 - 0.572)	0.398	0.048 (-0.182 - 0.277)	0.686	0.235 (-0.338 - 0.807)	0.421
HDCA	0.037 (-0.257 - 0.330)	0.807	0.016 (-0.150 - 0.182)	0.850	0.047 (-0.374 - 0.468)	0.828
LCA	0.028 (-0.261 - 0.316)	0.852	-0.104 (-0.267 - 0.060)	0.215	0.093 (-0.322 - 0.508)	0.660
MCA	0.386 (0.011 - 0.761)	0.044	0.132 (-0.078 - 0.342)	0.218	0.513 (-0.024 - 1.05)	0.061
MDCA	0.067 (-0.277 - 0.411)	0.704	0.025 (-0.177 - 0.227)	0.809	0.088 (-0.402 - 0.577)	0.726
UDCA	0.230 (-0.109 - 0.570)	0.184	0.038 (-0.159 - 0.235)	0.703	0.327 (-0.159 - 0.812)	0.187
Secondary non-conjugated	0.134 (-0.141 - 0.409)	0.341	0.013 (-0.142 - 0.169)	0.866	0.194 (-0.202 - 0.589)	0.337
GDCA	0.228 (-0.166 - 0.621)	0.256	-0.086 (-0.317 - 0.144)	0.462	0.385 (-0.175 - 0.945)	0.178
GHCA	-0.032 (-0.282 - 0.217)	0.799	-0.085 (-0.224 - 0.055)	0.234	-0.006 (-0.365 - 0.353)	0.972
GHDCA	0.176 (-0.25 - 0.602)	0.417	0.063 (-0.185 - 0.311)	0.620	0.233 (-0.375 - 0.840)	0.453
GLCA	-0.138 (-0.548 - 0.272)	0.509	**-0.374 (-0.613 - -0.135)**	**0.002**	-0.020 (-0.604 - 0.564)	0.947
GLCA-S	0.089 (-0.191 - 0.368)	0.533	-0.074 (-0.231 - 0.083)	0.356	0.170 (-0.232 - 0.572)	0.406
GUDCA	0.005 (-0.290 - 0.300)	0.973	-0.019 (-0.187 - 0.149)	0.827	0.017 (-0.406 - 0.440)	0.937
TDCA	0.125 (-0.277 - 0.528)	0.541	-0.146 (-0.382 - 0.090)	0.225	0.261 (-0.311 - 0.833)	0.371
THCA	0.071 (-0.360 - 0.502)	0.745	-0.040 (-0.282 - 0.202)	0.746	0.127 (-0.491 - 0.745)	0.687
THDCA	0.179 (-0.255 - 0.612)	0.419	-0.130 (-0.389 - 0.128)	0.322	0.333 (-0.283 - 0.949)	0.289
TLCA	0.071 (-0.287 - 0.429)	0.699	-0.161 (-0.369 - 0.047)	0.130	0.186 (-0.324 - 0.697)	0.474
TUDCA	0.056 (-0.309 - 0.420)	0.764	0.048 (-0.162 - 0.258)	0.651	0.059 (-0.463 - 0.581)	0.823
Secondary conjugated	0.159 (-0.072 - 0.389)	0.176	-0.100 (-0.228 - 0.028)	0.125	0.288 (-0.044 - 0.621)	0.089
Total bile acids	0.045 (-0.163 - 0.253)	0.670	-0.106 (-0.220 - 0.008)	0.069	0.121 (-0.180 - 0.421)	0.430

Table S10. Hazard ratios, including 95% confidence intervals and p-values for the Cox regression of bile acids association with reaching EDSS milestone 4, stratified based sex. A p-value ≤0.0125 was considered significant, significant p-values are highlighted with bold text.

	Males		***Females***	
	**HR (95% CI)**	**p-value**	**HR (95 % CI)**	**p-value**
CDCA	1.01 (0.839 - 1.22)	0.899	1.01 (0.907 - 1.12)	0.904
CA	0.979 (0.821 - 1.17)	0.811	1.02 (0.927 - 1.12)	0.708
Primary non-conjugated	0.993 (0.822 - 1.20)	0.940	1.02 (0.920 - 1.13)	0.699
GCDCA	1.19 (0.957 - 1.49)	0.117	**1.26 (1.06 - 1.50)**	**0.008**
GCA	1.13 (0.948 - 1.34)	0.176	1.19 (1.02 - 1.39)	0.029
TCDCA	1.17 (0.98 - 1.40)	0.083	1.16 (1.01 - 1.34)	0.032
TCA	1.15 (0.987 - 1.35)	0.072	1.10 (0.976 - 1.25)	0.114
Primary conjugated	1.15 (0.957 - 1.39)	0.133	**1.24 (1.05 - 1.48)**	**0.012**
DCA	1.05 (0.885 - 1.25)	0.574	1.07 (0.950 - 1.20)	0.279
HCA	0.929 (0.774 - 1.11)	0.427	0.999 (0.909 - 1.10)	0.988
HDCA	1.05 (0.848 - 1.30)	0.651	1.13 (0.987 - 1.28)	0.078
LCA	1.18 (0.908 - 1.52)	0.219	1.15 (1.01 - 1.31)	0.031
MCA	1.03 (0.853 - 1.25)	0.747	1.01 (0.897 - 1.13)	0.915
MDCA	1.18 (0.941 - 1.47)	0.153	1.06 (0.942 - 1.19)	0.332
UDCA	1.14 (0.948 - 1.37)	0.164	1.03 (0.903 - 1.17)	0.688
Secondary non-conjugated	1.07 (0.864 - 1.34)	0.518	1.08 (0.944 - 1.23)	0.271
GDCA	1.11 (0.925 - 1.33)	0.263	1.11 (0.986 - 1.25)	0.085
GHCA	1.17 (0.926 - 1.49)	0.184	1.17 (1.01 - 1.35)	0.032
GHDCA	**1.23 (1.05 - 1.44)**	**0.010**	1.03 (0.942 - 1.13)	0.506
GLCA	1.09 (0.933 - 1.28)	0.272	1.10 (1.00 - 1.22)	0.051
GLCAS	1.19 (0.937 - 1.50)	0.156	1.13 (0.999 - 1.28)	0.052
GUDCA	1.20 (0.963 - 1.49)	0.105	1.11 (0.967 - 1.28)	0.138
TDCA	1.12 (0.948 - 1.33)	0.180	1.09 (0.973 - 1.23)	0.133
THCA	1.23 (0.975 - 1.55)	0.081	1.05 (0.949 - 1.16)	0.338
THDCA	1.13 (0.973 - 1.31)	0.111	1.07 (0.961 - 1.19)	0.215
TLCA	1.12 (0.943 - 1.34)	0.190	1.15 (1.02 - 1.30)	0.023
TUDCA	1.25 (1.03 - 1.52)	0.025	1.02 (0.910 - 1.15)	0.701
Secondary conjugated	1.19 (0.951 - 1.49)	0.128	**1.23 (1.05 - 1.44)**	**0.009**
Total bile acids	1.17 (0.944 - 1.45)	0.151	1.25 (1.04 - 1.51)	0.015

Table S11. Hazard ratios, including 95% confidence intervals and p-values for the Cox regression of bile acids association with reaching EDSS milestone 6.

	HR (95% CI)	***p-value***
CDCA	1.03 (0.913 - 1.15)	0.662
CA	1.00 (0.887 - 1.13)	0.969
Primary unconjugated	0.999 (0.869 - 1.15)	0.986
GCDCA	1.08 (0.891 - 1.30)	0.443
GCA	1.15 (0.981 - 1.34)	0.085
TCDCA	1.07 (0.930 - 1.23)	0.342
TCA	1.11 (0.984 - 1.26)	0.087
Primary conjugated	1.12 (0.939 - 1.33)	0.213
DCA	1.08 (0.943 - 1.24)	0.259
HCA	0.980 (0.872 - 1.10)	0.740
HDCA	1.05 (0.898 - 1.23)	0.545
LCA	1.10 (0.932 - 1.29)	0.263
MCA	1.05 (0.920 - 1.19)	0.477
MDCA	1.03 (0.893 - 1.19)	0.665
UDCA	1.01 (0.863 - 1.18)	0.901
Secondary unconjugated	1.07 (0.908 - 1.26)	0.415
GDCA	1.09 (0.955 - 1.25)	0.198
GHCA	1.14 (0.953 - 1.37)	0.149
GHDCA	1.04 (0.946 - 1.15)	0.388
GLCA	1.01 (0.913 - 1.12)	0.812
GLCAS	1.07 (0.918 - 1.24)	0.404
GUDCA	1.09 (0.910 - 1.30)	0.350
TDCA	1.11 (0.973 - 1.25)	0.123
THCA	1.13 (0.990 - 1.29)	0.067
THDCA	1.06 (0.938 - 1.20)	0.339
TLCA	1.07 (0.937 - 1.23)	0.302
TUDCA	1.05 (0.916 - 1.20)	0.489
Secondary conjugated	1.13 (0.944 - 1.35)	0.183
Total bile acids	1.15 (0.942 - 1.41)	0.168
